# Tuning the superconducting performance of YBa_2_Cu_3_O_7−δ_ films through field-induced oxygen doping

**DOI:** 10.1038/s41598-024-52051-1

**Published:** 2024-01-22

**Authors:** Jordi Alcalà, Alejandro Fernández-Rodríguez, Thomas Günkel, Aleix Barrera, Mariona Cabero, Jaume Gazquez, Lluis Balcells, Narcís Mestres, Anna Palau

**Affiliations:** 1 Institut de Ciència de Materials de Barcelona, ICMAB-CSIC, Campus UAB, 08193 Bellaterra, Barcelona Spain; 2 IMDEA Nanoscience Institute, Campus Universidad Autonoma, 28049 Madrid, Spain; 3 Centro Nacional de Microscopia Electrónica, Universidad Complutense, 28040 Madrid, Spain

**Keywords:** Superconducting properties and materials, Electronic properties and materials

## Abstract

The exploration of metal–insulator transitions to produce field-induced reversible resistive switching effects has been a longstanding pursuit in materials science. Although the resistive switching effect in strongly correlated oxides is often associated with the creation or annihilation of oxygen vacancies, the underlying mechanisms behind this phenomenon are complex and, in many cases, still not clear. This study focuses on the analysis of the superconducting performance of cuprate YBa_2_Cu_3_O_7−δ_ (YBCO) devices switched to different resistive states through gate voltage pulses. The goal is to evaluate the effect of field-induced oxygen diffusion on the magnetic field and angular dependence of the critical current density and identify the role of induced defects in the switching performance. Transition electron microscopy measurements indicate that field-induced transition to high resistance states occurs through the generation of YBa_2_Cu_4_O_7_ (Y124) intergrowths with a large amount of oxygen vacancies, in agreement with the obtained critical current density dependences. These results have significant implications for better understanding the mechanisms of field-induced oxygen doping in cuprate superconductors and their role on the superconducting performance.

## Introduction

Today advances to store and analyse massive information offer huge opportunities and unprecedented benefits in business, healthcare, security, or society, but require to explore novel technologies for information storage and processing in a sustainable way. Among many different approaches, strongly correlated oxides are particularly interesting materials for emerging electronic devices due to their rich phase diagram with transitions between competing phases showing dramatically different electronic and magnetic properties^1^. In particular, metal–insulator transitions (MITs) can be exploited to induce a non-volatile reversible switch between different resistance states induced by an electric field^2^. In simple terms, resistive switching (RS) devices based on strongly correlated oxides display significant resistance variations caused by small carrier concentration modulations driven by an electric field, enabling the stabilization of non-volatile multilevel analogic states^3^. Although the underlying mechanisms behind RS phenomena are complex and dependent on the specific materials involved, in oxide systems, the switching behaviour is often associated with the movement of oxygen vacancies^4^. This movement can manifest as a metallic filament embedded within an insulating matrix^4^, modification of a Schottky barrier at the contact interface^5^, or due to a homogeneous switch through the material volume^6–9^.

Particularly interesting is the modulation of the non-volatile MIT in cuprate superconductors YBa_2_Cu_3_O_7−δ_ (YBCO) where both the normal state resistance and the superconducting critical temperature can be reversibly manipulated in confined active volumes by field-induced electrochemical oxygen doping^10,11^ or electromigration effects^12–15^. Fine tuning of the oxygen doping through voltage pulses of electric current allow to explore the electrical transport properties of complex phases appearing in the doping phase diagram of cuprates^10,13,15^.

In this work we use field-induced resistive switching effects to study the critical current density performance of YBCO films at different oxygen doping levels. The effect of oxygen doping on the magnetic field and angular dependence of the critical current density may be of great relevance for the optimization of flux pinning through microstructural modification^16–18^. Here we are able to selectively tune the doping level of superconducting tracks by applying voltage pulses. The effect of field-induced oxygen doping is studied by measuring the critical temperature, carrier density, critical current density at different resistance levels. Transition electron microscopy experiments performed at low and high resistive states indicate the generation of a large amount of YBa_2_Cu_4_O_7_ (Y124) intergrowths with a high density of oxygen vacancies. A clear correlation between the microstructure with the magnetic field and angular dependencies of the critical current density is obtained.

## Methods

### Sample fabrication

**Figure 1:**
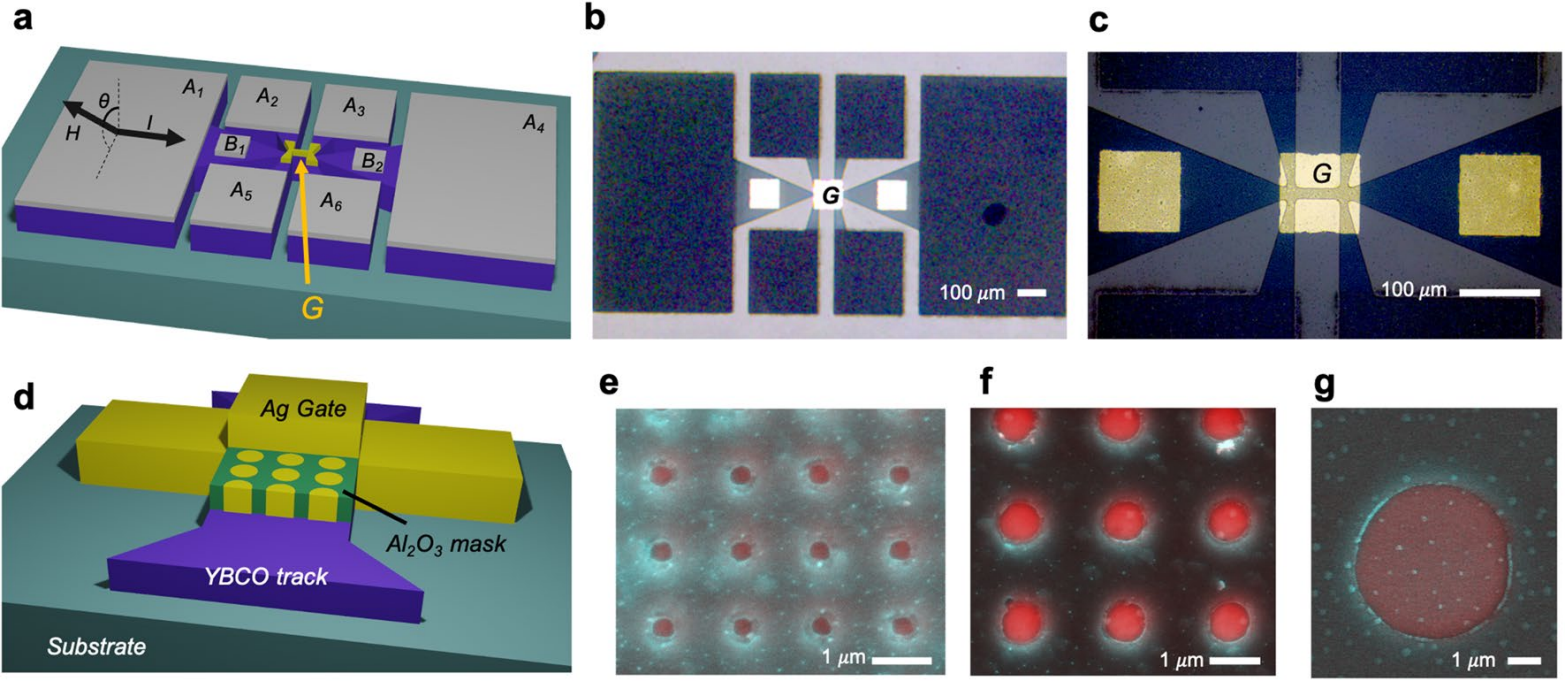
YBCO devices patterned with different gates. (**a**) Schematic representation of the measurement configuration. Gate contact (G) used to induce the switching effect is depicted in yellow (**b,c**) Optical images of a YBCO device with a uniform gate, G, of 100 µm × 100 µm, covering all the YBCO track. (**d**) Scheme of gate preparation using an insulating mask. (**e–g**) Masks with different dot size.

Epitaxial YBa_2_Cu_3_O_7−δ_ (YBCO) thin films of thicknesses ranging from 50 to 250 nm were grown by pulsed laser deposition (PLD) on (001)-LaAlO_3_ (LAO) or SrTiO_3_ (STO) single crystal substrates. Substrates were heated up to T = 800–810 °C, with an O_2_ partial pressure of 0.3 mbar during the deposition and a fixed target-substrate distance of 52.5 mm. A high fluence laser (around 2 J/cm^2^) working at a frequency of 5 Hz was used. During the cooling ramp, we increase the P(O_2_) in the chamber in order to obtain well oxygenated samples. The thickness of the film is mainly determined by the number of pulses considering 26 pulses for 1 nm and checked in the profilometer. For these samples, we obtain thickness of 50, 100 and 250 nm using 1300, 2600 and 6500 pulses, respectively. YBCO devices were patterned with specially designed contact paths enabling the modulation of oxygen doping in a localized track through a gate contact (*G* in Fig. 1a). Photolithography combined with sputtering and lift-off were used to deposit 200 nm thick silver contacts on top of large paths which were used to apply current (*A*_1_ and *A*_4_ of 500 µm × 850 µm) and measure longitudinal and transverse voltage (*A*_2_,* A*_3_,* A*_5_ and* A*_6_ contacts, of 250 µm × 300 µm). In order to assure good contact resistance, samples were annealed at 450 °C during 1 h. Photolithography and wet etching were used to pattern YBCO tracks of width *w* = 30 μm and length *l* = 100 μm. After the patterning, 50 nm thick small silver contacts (*B*_1_,* B*_2_) of 100 µm × 100 µm were deposited by sputtering and lift-off. Finally, a gate contact (*G*) was deposited on top of the YBCO track. Figure 1b,c shows optical images of one of the measured devices with a top gate of 100 µm × 100 µm. Additionally, we fabricated gates with different areas by using an intermediate insulating mask of Al_2_O_3_ of 30 nm which was patterned by Electron Beam Lithography with an array of dots of the desired dimension (Fig. 1d). A top 50 nm silver contact of 300 µm × 100 µm was deposited by sputtering on top of the patterned Al_2_O_3_. This contact was deported away from the track to avoid damaging of the patterned insulating buffer layer while bounding the sample. Figure 1e–g show scanning electronic images of Al_2_O_3_ masks patterned with dots of different dimensions.

### Superconducting properties

Transport measurements were performed using a four-probe configuration using a Quantum Design physical property measurement system (PPMS). Field dependence and angular curves of the critical current density, *J*_*c*_*(H)*, *J*_*c*_*(θ)*, were obtained at 77 K and 85 K using an electric field criterion of 2 µV cm^−1^. *J*_*c*_*(H)* was measured with the magnetic field parallel to the *c*-axis, *H//c*. The rotation angle, *θ,* was changed from − 60° to 190°, being *θ* = 90° magnetic field parallel to the *ab-*planes. The crossover magnetic field from single vortex pinning to collective pinning regimes, H*, was determined at 90% of self-field *J*_*c*_. Error bars in *J*_*c*_ and H* have been determined considering a 10% deviation from the criterion. The width of the *J*_*c*_*(θ) H//ab* peak, Δ*θ*, was obtained as half of the Full Width at Half Maximum of Lorentzian fits. Error bars are standard deviation to those fits. Critical temperature, *T*_*c*_, was determined from the resistance vs. temperature curves, *R*(*T*) at the onset of zero resistance with a 10^–3^ Ω threshold criterion. Error bars were estimated considering the temperature resolution at the criterium. Carrier density was calculated using *n* = 1/*R*_*H*_*e*, being* e* the electron charge and *R*_*H*_ = (*t*/*I*)d*V*_*H*_/d*B* the Hall coefficient, *t* the sample thickness, *I* the applied current, and d*V*_*H*_/d*B* the linear slope of the Hall voltage with the magnetic field. Error bars in *n* were calculated from the linear fit standard deviation. The oxygen modulation was carried out through different sweep voltages that were applied between two top gates (top-top configuration), at room temperature with a Keithley 2450 source-meter. Sweep voltages were performed applying 50 steps from to 0 V to the desired voltage, and 50 steps from this last value back to 0 V (100 points in total), being the time width of each step 0.11 s and the total time for a complete loop of 11 s.

### Scanning transmission electron microscopy characterization

Aberration-corrected scanning transmission electron microscopy (STEM) was used for microstructural analysis with atomic resolution. Samples were characterized using a JEOL JEM ARM200cF operated at 200 kV, equipped with a CEOS aberration corrector and GIF Quantum ER spectrometer, at the Universidad Complutense de Madrid, Spain. The STEM images were acquired in high angle annular dark field imaging mode, also referred to as Z-contrast because the brightness associated to each atomic column roughly scales with the square of the atomic number Z^19^. The STEM specimens were prepared using a FEI Helios nanolab 650, at SEM–FIB microscopy service of the Universidad de Málaga, Spain.

## Results and discussion

### Modulation of the room temperature gate resistance state

**Figure 2:**
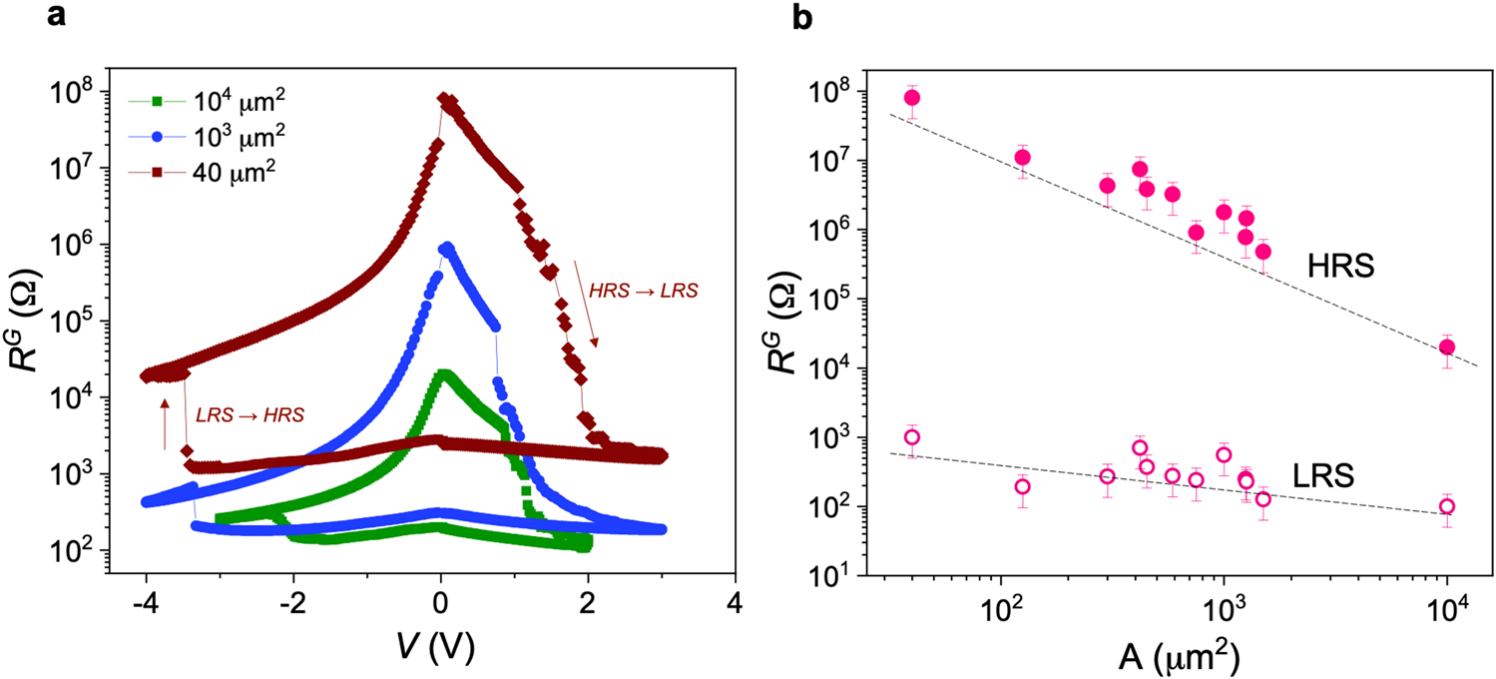
Bipolar resistive switching loops of YBCO devices. (**a**) *R*^*G*^*-V* characteristics of YBCO devices with different gate size. Arrows show the voltage sweep direction in one of the hysteresis loops. (**b**) Evolution of HRS and LRS, obtained measuring at 0.1 V, as a function of the contact area. Error bars correspond to variation of resistance state after different hysteresis loops.

We first study the resistive switching performance of the YBCO devices at room temperature by applying voltage pulses at the gate *G* by using one of the lateral contacts *B*1 or *B*2 to measure the gate resistance in a two-point configuration, *R*^*G*^. Figure 2a shows several *R*^*G*^*-V* curves obtained in different devices by changing the size of the gate contact. Non-volatile bipolar resistive switching behaviour is found where a transition from a low resistance state (LRS) to a high resistance state (HRS) occurs by applying a negative voltage. The system can be reversibly switched back to the LRS by applying positive voltage. The main mechanism responsible for the resistance change is the oxygen doping modulation (tunning of δ in YBa_2_Cu_3_O_7−δ_) through field-induced oxygen diffusion^10^. The obtained hysteresis loops are asymmetric with a higher voltage needed to drive the system from the LRS to the HRS than that required to go from the HRS to the LRS, as theoretically predicted considering an asymmetry in the oxygen motion^10^. It is also observed that in general, a progressive resistance change from HRS to LRS is obtained when oxygen is incorporated to the system while a fast transition is produced by oxygen migration. Figure S1, Supporting Information, shows three consecutive *R–V* hysteresis curves, indicating the reversibility characteristics of the switching effect. It should be noted that the hysteresis is strongly increased by decreasing the gate area as expected for a volume metal-insulating transition homogeneously occurring below the gate^10^. Very high resistance ratio up to 10^5^ can be obtained in devices with a gate area of 40 μm^2^ with a relatively low operation voltage ~ 2–3 V. The resistance ratio, particularly important for device applications, should be further increased by reducing the gate dimension at nanoscale.

### Modulation of the superconducting performance

**Figure 3:**
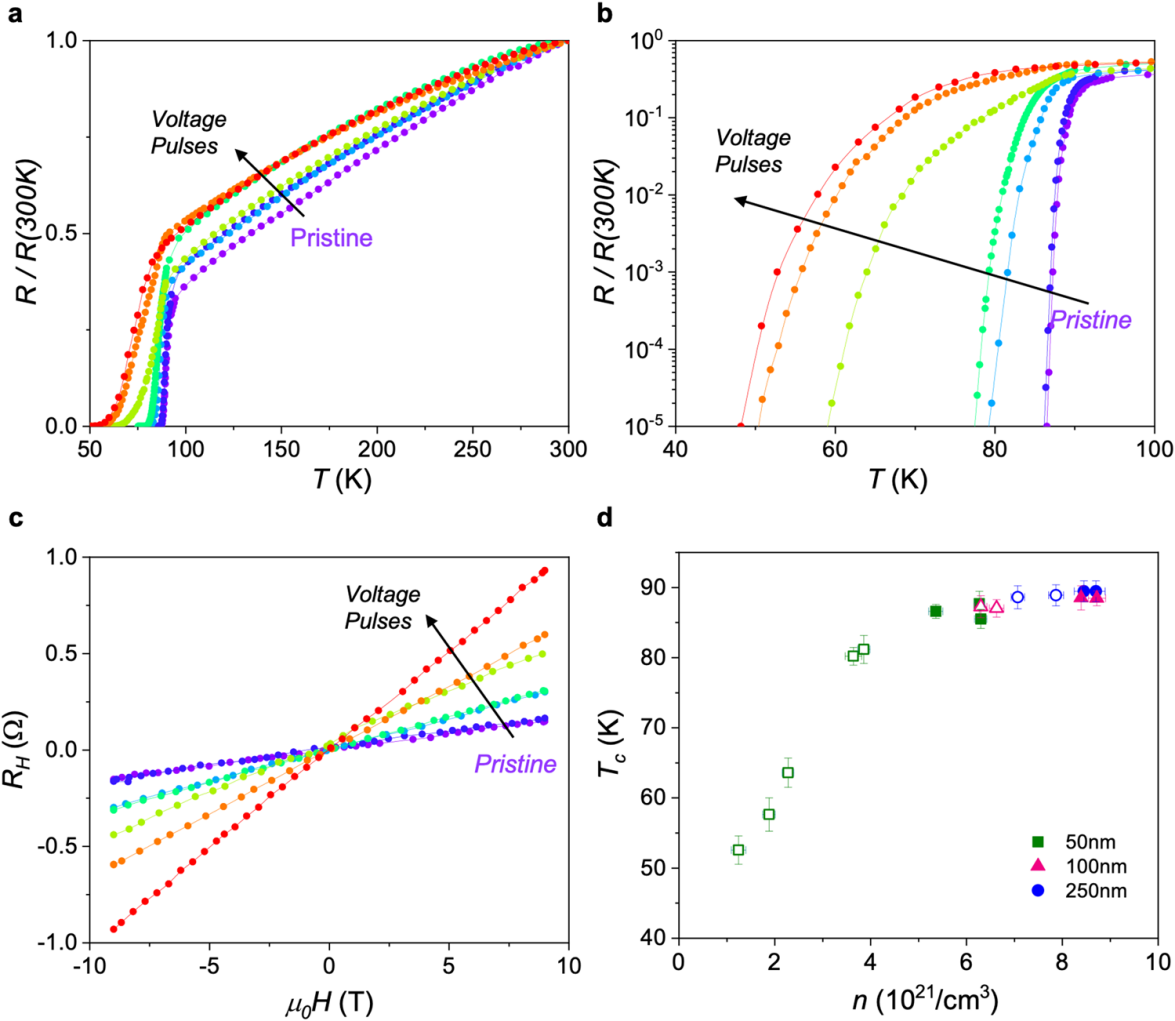
Critical temperature and carrier density at different oxygen doping levels. (**a**) Linear and (**b**) log-scale normalized resistance as a function of temperature, for a 50 nm sample at the pristine LRS and after several consecutive voltage switches at − 1.8 V, − 2 V, − 2.5 V, − 3 V, − 4 V and − 5 V. (**c**) Evolution of the Hall resistance vs magnetic field at 300 K with the applied voltage switch. (**d**) Superconducting critical temperature, *T*_*c*_, as a function of the carrier density, *n*, obtained for YBCO tracks of different thickness at the LRS (closed symbols) and after applying several negative voltages switches at different HRS (open symbols). Green squared points correspond to samples shown in (**a–c**).

In order to elucidate the effect of field-induced oxygen doping in the superconducting properties, we have studied the magnetic field and angular dependence of the critical current density, *J*_*c*_, at different resistance states (doping levels), and correlate it with microstructural changes induced by the switching process. To do so, voltage pulses were applied at 300 K on patterned YBCO films of different thicknesses, using a large gate contact of A = 100 µm × 100 µm covering the whole track (Fig. 1b,c). After each switch we measured the change in the volume resistance and carrier density of the track using transversal and lateral voltage contacts (see “Methods” section). Figure 3 shows that a systematic decrease of* T*_*c*_ and increase of *R*_*H*_ (decrease of the carrier density, *n*) is obtained when applying successive negative voltage pulses to the 50 nm sample inducing transitions to different HRS levels. Figure 3c shows *T*_*c*_ as a function of *n* for samples with different thickness in the pristine LRS state (closed symbols) and after being switched to different high resistance states (open symbols). A continuous modulation of the carrier density to different underdoped states and associated *T*_*c*_ reduction is observed for the thinnest sample (*t* = 50 nm). Many different resistance levels may be obtained by changing the applied gate voltage. As expected, the variation obtained for thicker samples is much lower since in this case not all the track thickness under the gate has been switched to the high resistance state^10^. *T*_*c*_ and *R*_*H*_ plots obtained for the 100 nm and 250 nm thick samples are shown at the Supporting Information (Figs. S2, S3). As it will be discussed below, reversible and irreversible resistance changes can be obtained, depending on the movement and redistribution of oxygen vacancies within the track and/or the generation of defects.

**Figure 4:**
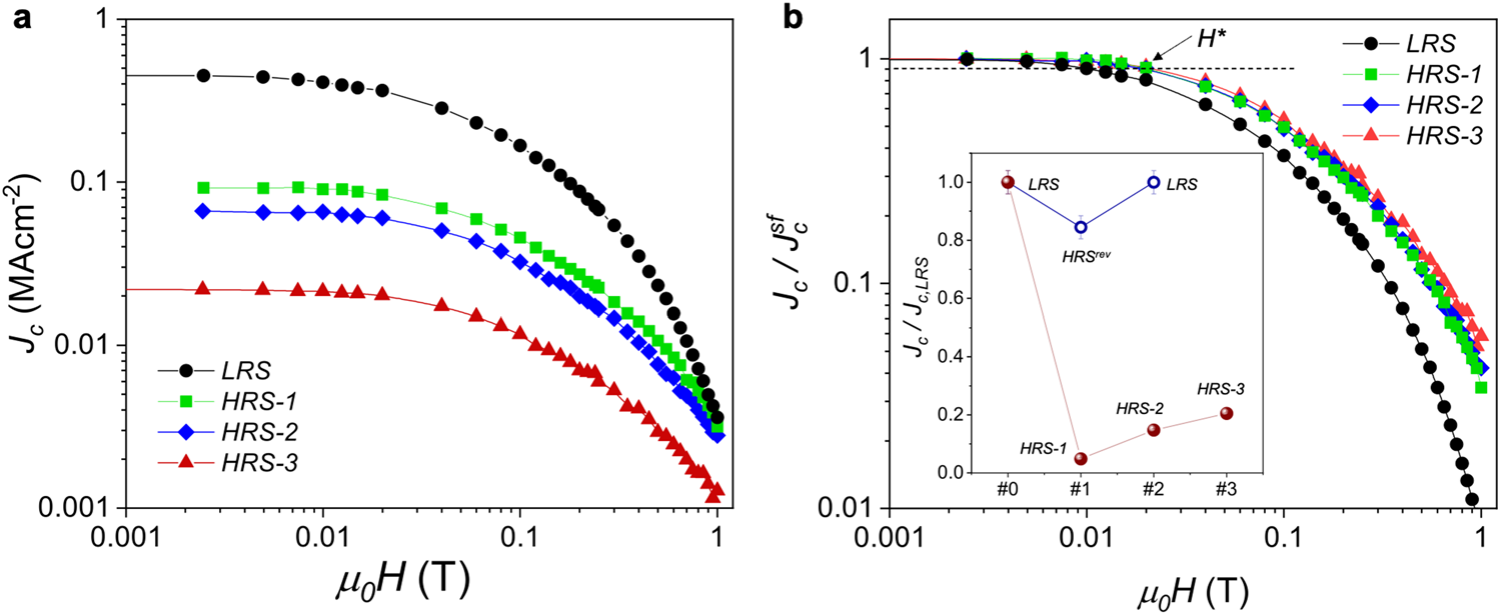
Field dependence of the critical current density. (**a**) *J*_*c*_(*H*) curves measured at 1 T *H//c*, 85 K, for a 100 nm sample at three different oxygen doping states (**b**) *J*_*c*_(*H*) curves shown in (**a**) normalized at the value of self-field*, J*_*c*_^*sf*^. Inset in (**b**) shows the evolution of *J*_*c*_ after being switched to different irreversible HRS using high voltage pulses (red symbols), and reversible HRS^rev^ at low voltage pulses (blue symbols).

**Figure 5:**
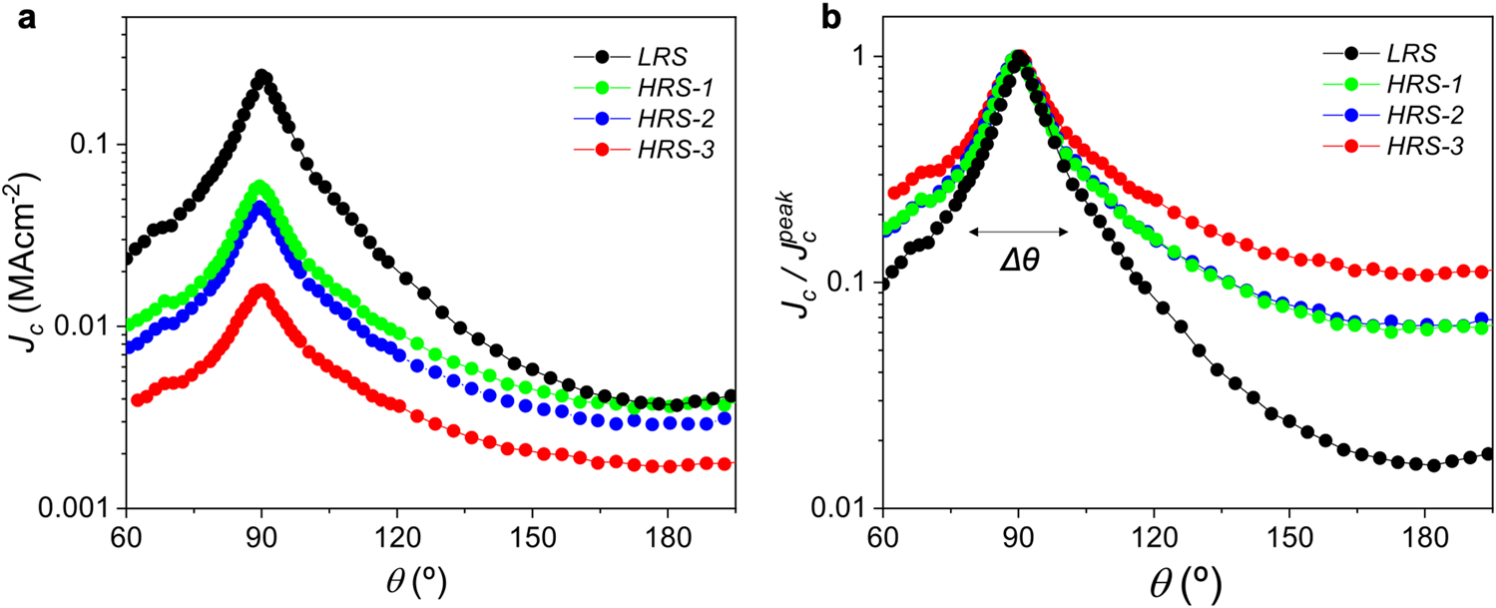
Angular dependence of the critical current density. (**a**) *J*_*c*_*(θ)* curves measured at 1 T, 85 K for a 100 nm sample at three different oxygen doping states. (**b**) *J*_*c*_*(θ)* curves shown in (**a**) normalized at the maximum value of peak at *H//ab* (*θ* = 90°).

The fine control of the oxygen content with the switching voltage allows us to perform a systematic study of the critical current density in the YBCO track, at different resistance states. Figure 4a and 5a shows the magnetic field and angular dependence, respectively, of *J*_*c*_ for a 100 nm sample at the pristine state and after being switched to three different HRS. Starting from a pristine LRS we switch the sample to HRS-3 by applying − 4 V. A very strong decrease of *J*_*c*_ of more than one order of magnitude is observed. The track can be partially recovered to intermediated high resistance states by inverting the voltage polarity (HRS-2 @ 3 V and HRS-3 @4 V), although the system cannot be reversibly switched back to the initial LRS. Nevertheless, we observed that *J*_*c*_ can be switched to intermediate reversible high resistance states, HRS^rev^, if oxygen-depleted states are induced by applying lower voltage pulses. In this case the modulation of *J*_*c*_ may be kept below 20% (Inset in Fig. 4b).

**Figure 6:**
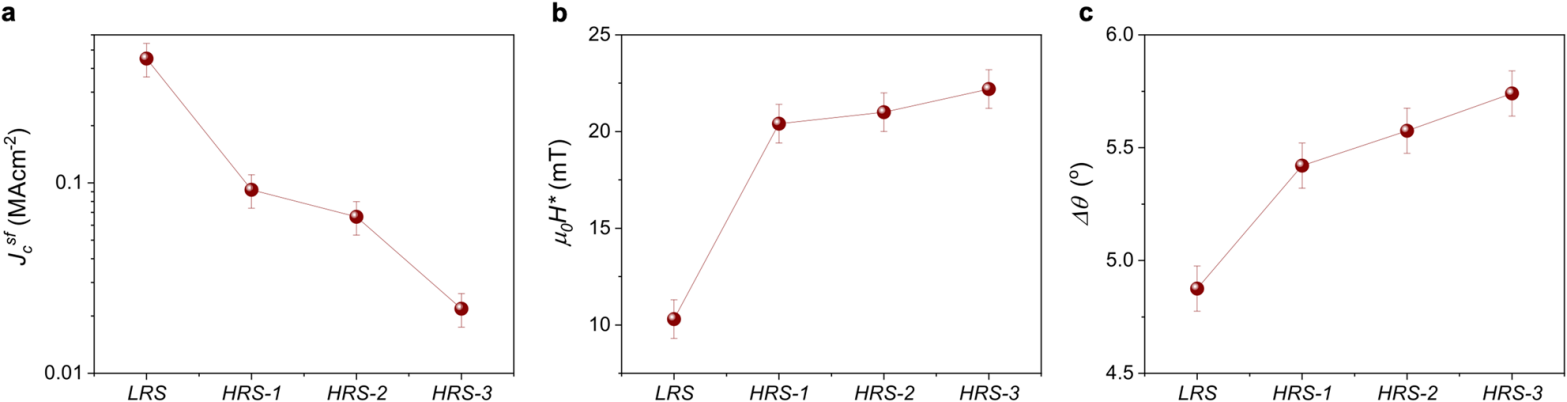
Dependence of the superconducting performance with oxygen doping level. Evolution of (**a**) self-field critical current density, *J*_*c*_^*sf*^, (**b**) crossover magnetic field from a single vortex pinning to collective pinning regime, H*, and (**c**) width of the *H//ab* peak, Δ*θ*, by reducing the oxygen doping level through gate voltage pulses.

The changes in *J*_*c*_(*H*) and *J*_*c*_*(θ)* at different doping levels can be better appreciated in the normalized plots shown in Figs. 4b and 5b, respectively. Overall, we observe a smaller magnetic field decay of *J*_*c*_ with an associated shift of H* to higher fields, and a broadening of the *H//ab* peak by switching the sample to HRS. The evolution of the *J*_*c*_^*sf*^, H* and Δ*θ* with the reduction of the oxygen doping level are depicted in Fig. 6.

**Figure 7:**
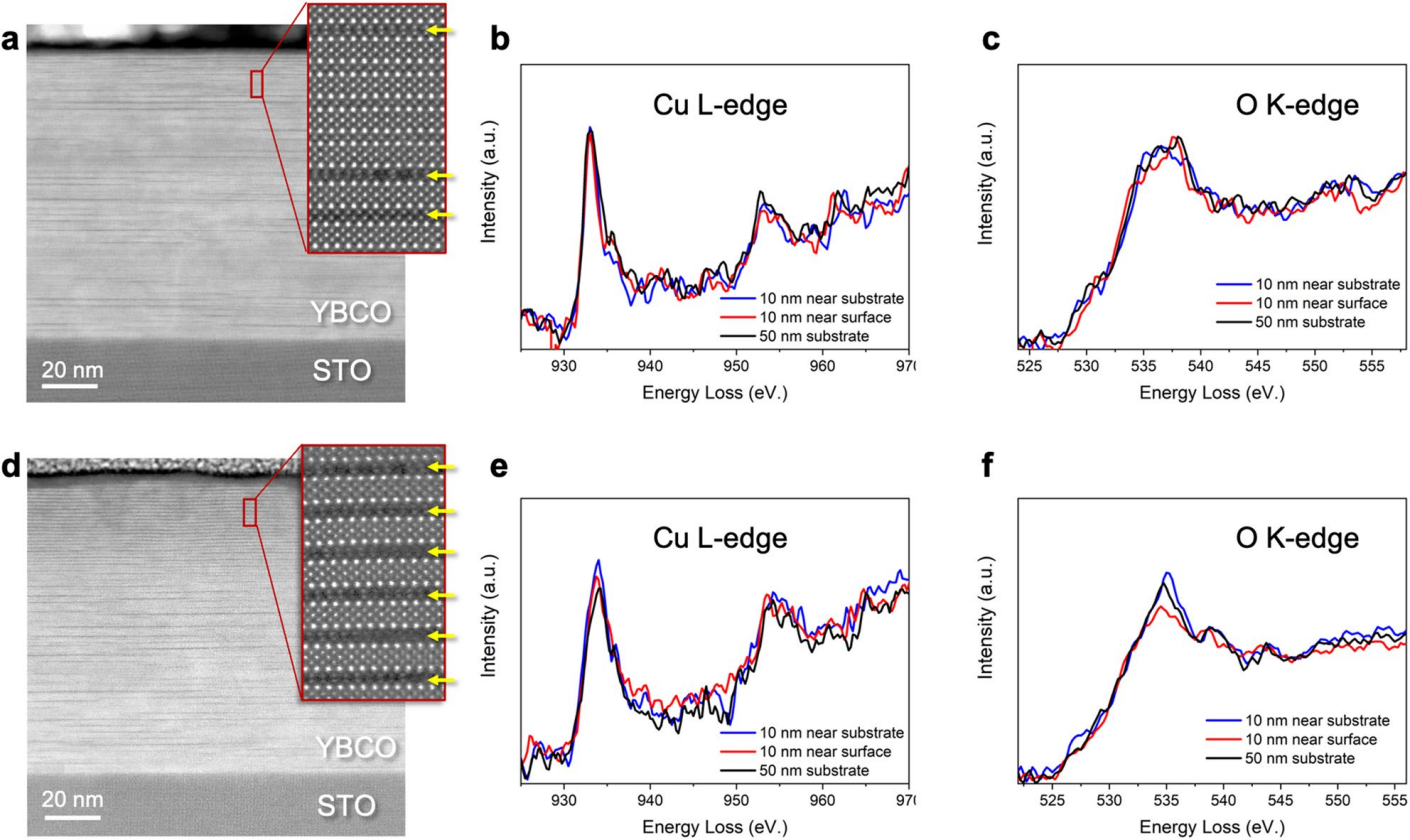
Microstructural defects. (**a**) Z-contrast image of a 100 nm YBCO track at the LRS, (**b**) Cu L-edge, and (**c**) O K-edge acquired at different regions of the pristine track. (**d**) Z-contrast image of a YBCO track switched to the HRS after applying a voltage pulse of − 4 V. (**e**) Cu L-edge and (**f**) O K-edge at the HRS acquired at different regions of track. Insets in (**a,d**) show a high-resolution Z-contrast image of the regions marked in red.

In order to elucidate the defects that produce a change in the *J*_*c*_ performance at the HRS, the microstructure of the YBCO layer was studied using scanning transmission electron microscopy (STEM) in combination with electron energy-loss spectroscopy (EELS). Figures 7a,d show a Z-contrast image of a YBCO track in the LRS and HRS, respectively. Both images show an epitaxial and coherent YBCO layer as well as dark stripes running parallel to the (001) YBCO plane. These planar defects are the common and well known as YBa_2_Cu_4_O_7_ (Y124) structural defects, which consist of an intergrowth of an extra Cu–O chain layer inserted into the YBCO^20^. It should be noted, however, that sample switched to the HRS presents a higher density of Y124 intergrowths, especially at the uppermost region of the layer. Indeed, close to the surface of the film there is an around 40 nm thick layer of pure Y124 phase, which is absent in the YBCO layer when it is the LRS. Insets in Fig. 7a,d show atomic-resolution images of the highlight regions, in which the arrows point at the double Cu–O chain.

We also studied the electronic structure of the YBCO layer at high and low resistance states by means of EELS. Figure 7b,c,e,f show the Cu L and the O K edges at the LRS and HRS, respectively, acquired in different regions of the YBCO layer. Notice the different fine structure or shape of the center peak (535 eV) of the O-K edge, or the different L3/L2 intensity ratio of the Cu L-edge when the YBCO layer is found at different resistance states. These differences are consistent with the presence of a higher concentration of oxygen vacancies and reduced Cu atoms in the HRS, which is also consistent with a larger number of double Cu–O chains, and therefore more reduced Cu atoms^21,22^.

TEM results indicate that the field-induced transition to the HRS occurs due to the formation of Y124 intergrowths with a high density of oxygen vacancies which can be clearly correlated with the obtained superconducting performance. A shift of H* to high fields together with a broadening of the *H//ab* peak observed when the sample is switched to the HRS (Fig. 6) is typically obtained in YBCO nanocomposites with a large amount of Y124 intergrowths induced by the presence of randomly oriented nanoparticles^23,24^. The formation of these large number of defects might be responsible for the non-reversible switching performance obtained when the system is driven to a very high oxygen-depleted state. According to our previous works, reversible effects are obtained when oxygen diffusion can be reversibly controlled homogeneously tunning the sample doping^10,11^. Irreversible effects, which may be associated to the presence of defects or inhomogeneous distributions of oxygen vacancies appear when thermal effects start to be important^12,15^. Indeed, thermal defect nucleation at large applied voltages, producing irreversible resistivity changes, have also been obtained in Perovskites^25^.

## Conclusions

We have studied the effect of field-induced metal insulating transition in YBCO devices on both the normal resistance state and superconducting performance. Our results demonstrate that reversible gate resistive switching effects, with very large resistive ratios up to 10^5^, can be obtained by applying moderate voltage pulses of 2–4 V using micrometric gate contacts. Gate voltage pulses have been used to tune the oxygen content of YBCO tracks and strongly manipulate the associated superconducting properties, including critical current temperature, carrier density and critical current density. Reversible changes in the superconducting performance may be obtained by inducing changes from LRS to HRS^rev^ with moderate oxygen-depleted regions. TEM measurements in agreement with *J*_*c*_*(H)*, *J*_*c*_*(θ)* dependencies indicate that the volume switch from LRS to different irreversible high oxygen-depleted high resistance states occurs through formation of Y124 intergrowths with a high density of oxygen vacancies. These results provide insight on understanding the role of field-induced defects in the switching performance of cuprate superconductors.

### Supplementary Information

Supplementary Figures.

## Data Availability

The data that support the findings of this study is available from the corresponding author upon request.
