# Supplementary material for: Tuning the superconducting performance of YBa2Cu3O7−δ films through field-induced oxygen doping
Source: Sci Rep. 2024 Jan 22;14:1939. doi: 10.1038/s41598-024-52051-1 (PMC10803336; doi:10.1038/s41598-024-52051-1)
Supplement: Supplementary file 1 — Supplementary Figures. [file 41598_2024_52051_MOESM1_ESM.docx]

Tuning the superconducting performance of YBa_2_Cu_3_O_7-δ_ films through field-induced oxygen doping

Jordi Alcalà* ^1^, Alejandro Fernández-Rodríguez ^1^, Thomas Günkel ^1^, Aleix Barrera ^1^, Mariona Cabero^2^, Jaume Gazquez^1^, Lluis Balcells ^1,^ Narcís Mestres ^1^, and Anna Palau* ^1^

^1^Institut de Ciència de Materials de Barcelona, ICMAB-CSIC, Campus UAB, 08193 Bellaterra, Barcelona, Spain; *palau@icmab.es, *jalcala@icmab.es

^2^ IMDEA Nanoscience Institute. Campus Universidad Autonoma, 28049, Madrid, Spain and Centro Nacional de Microscopia Electrónica. Universidad Complutense, 28040, Madrid, Spain


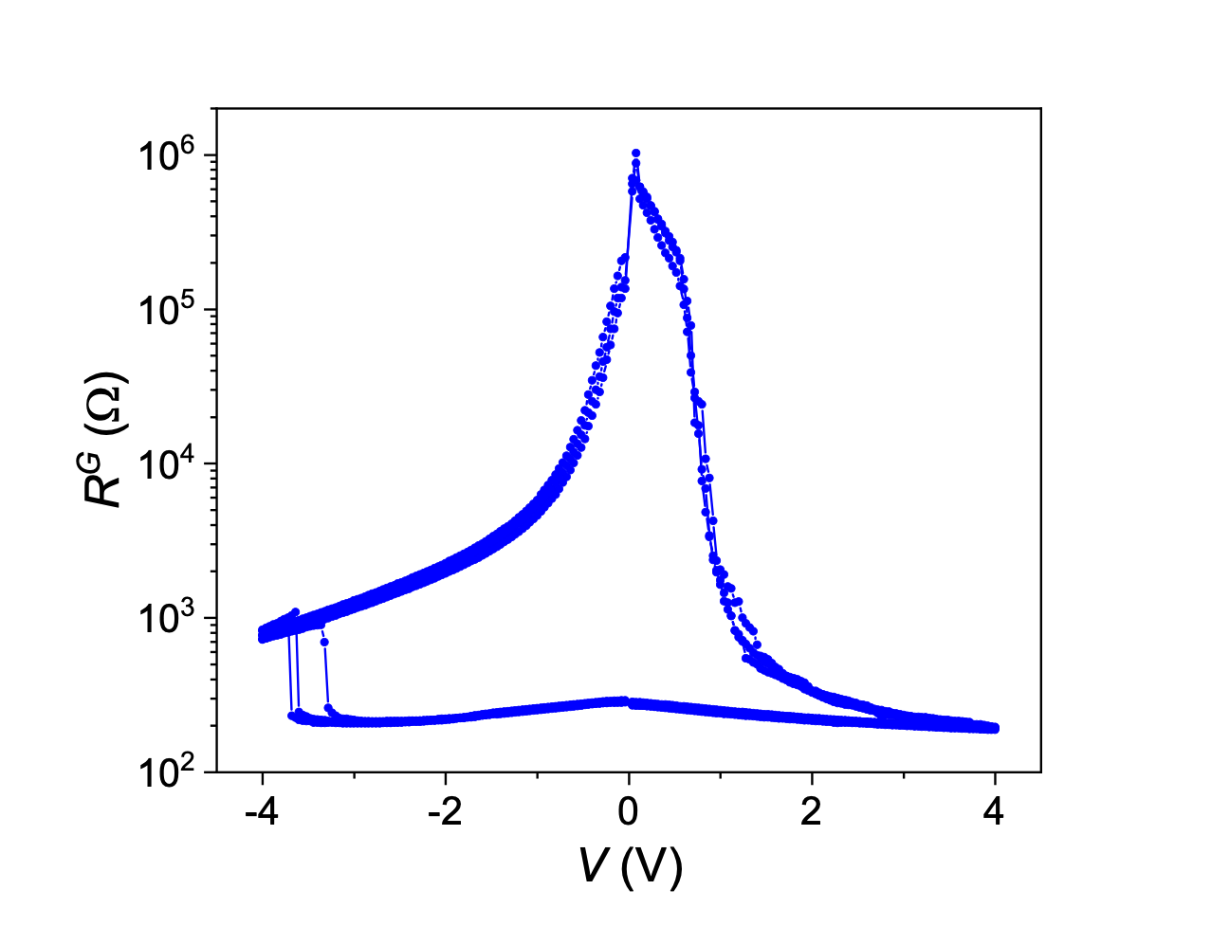


**Figure S1**: **Resistive switching loops at the gate contact.**  Gate resistance versus applied voltage obtained after several positive and negative voltage pulses showing the reversibility behaviour of the switching effect.


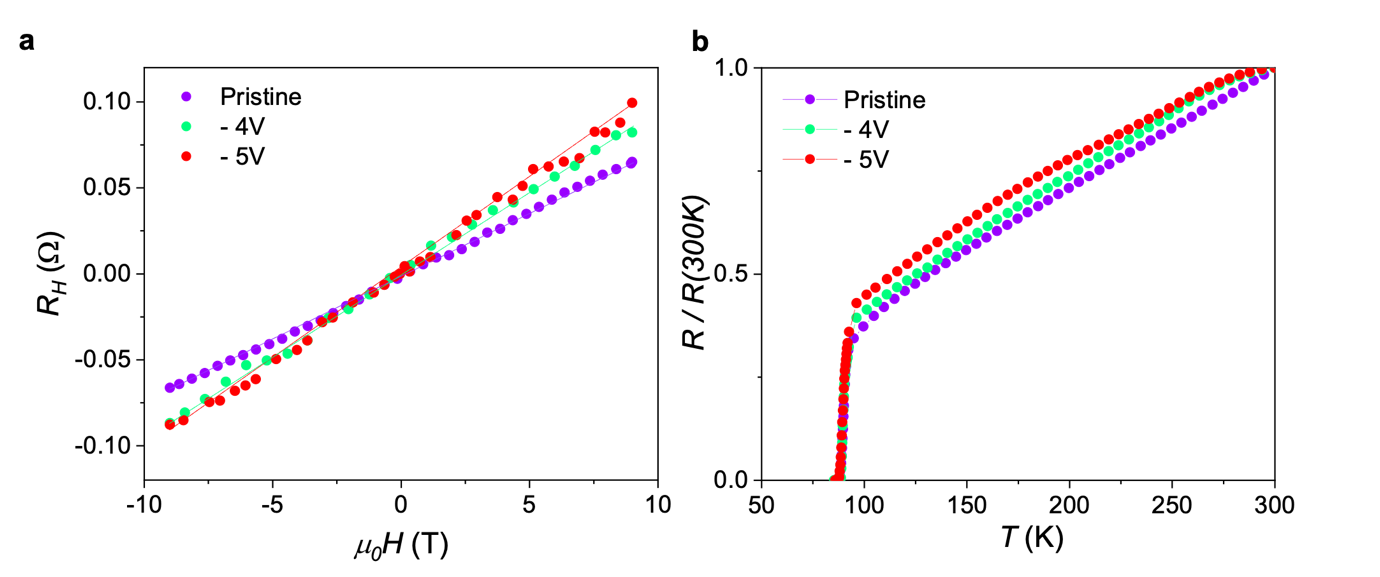


**Figure S2**: **Critical temperature and carrier density for a 100 nm YBCO sample.** Evolution of (a) Hall Resistance vs. applied magnetic field and (b) longitudinal resistance as a function of temperature for a 100nm YBCO sample at the pristine LRS and after several consecutive voltage switches.

**
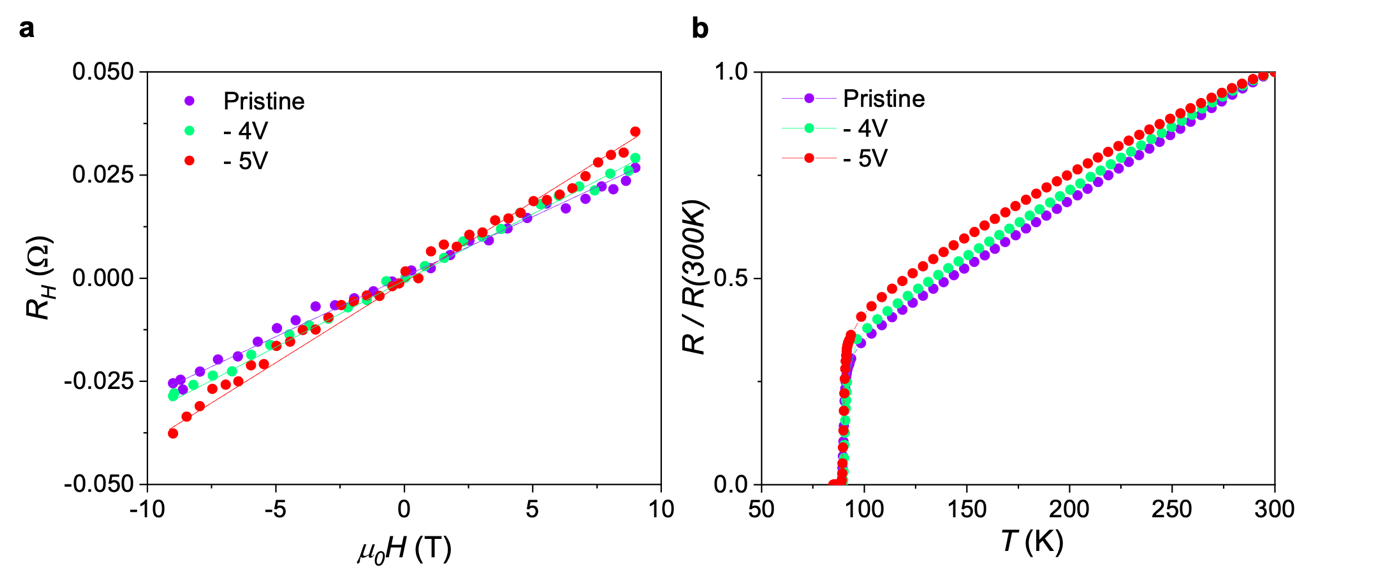
**

**Figure S3**: **Critical temperature and carrier density a 250 nm YBCO sample.** Evolution of (a) Hall Resistance vs. applied magnetic field and (b) longitudinal resistance as a function of temperature for a 250nm YBCO sample at the pristine LRS and after several consecutive voltage switches.
